# Serum HOTAIR as a novel diagnostic biomarker for esophageal squamous cell carcinoma

**DOI:** 10.1186/s12943-017-0643-6

**Published:** 2017-04-04

**Authors:** Wenjian Wang, Xiaotian He, Zehua Zheng, Xiaofan Ma, Xueting Hu, Duoguang Wu, Minghui Wang

**Affiliations:** Guangdong Provincial Key Laboratory of Malignant Tumor Epigenetics and Gene Regulation, Department of Thoracic surgery, Sun yat-sen memorial hospital, Sun yat-sen University, NO.107 of Yanjiangxi Road, Guangzhou, 510120 China

**Keywords:** Esophageal squamous cell carcinoma, Long noncoding RNA, HOTAIR, Biomarker, Diagnosis

## Abstract

**Background:**

Early diagnosis of esophageal squamous cell carcinoma (ESCC) is an important issue to improve the prognosis. HOX transcript antisense RNA (HOTAIR), a long noncoding RNA (lncRNA) expressed from the HOXC locus, has been recently revealed as an oncogenic regulator in ESCC. This study aimed to investigate whether serum HOTAIR is involved in the diagnosis of ESCC.

**Methods:**

In this study, we detected serum HOTAIR expression in 50 patients with ESCC (including 42 tumor resection and 8 without surgery) and 20 healthy volunteers to investigate the role of serum HOTAIR in ESCC using the quantitative real-time polymerase chain reaction (qRT-PCR) method.

**Results:**

Clinical data indicated that serum HOTAIR were correlated with TNM stage. The expression level of serum HOTAIR (0.189 ± 0.010) was significantly higher in ESCC patients compared with that of healthy controls (0.055 ± 0.008*, P* < 0.01). The ROC curve analysis yielded an area under the ROC curve (AUC) value of 0.793 (95% CI: 0.692 to 0.895, *P* < 0.01). Also, the serum HOTAIR expression level decreased obviously in postoperative samples (one month after the surgery) compared to preoperative specimens. Moreover, there was a significant correlation between serum HOTAIR expression and the expression of HOTAIR in ESCC tissue according to *Pearson* correlation analysis.

**Conclusions:**

Our study, for the first time, demonstrated that serum HOTAIR might serve as a potential biomarker for the diagnosis of ESCC.

## Introduction

Esophageal squamous cell carcinoma (ESCC) is the major histopathological subtype of esophageal cancer [1, 2]. Due to its tumor aggressiveness and therapeutic difficulties, ESCC ranks eighth in incidence and sixth in cancer death worldwide [3]. Approximately, 300,000 people die from esophageal cancer in the world annually [4]. Although the advances in medical and surgical treatments, most ESCC patients are diagnosed at an advanced stage with a poor prognosis, an overall five-year survival ranging from 15–25% [5]. Therefore, early detection of primary tumors provides effective treatments and timely interventions, which may improve the outcomes. To date, carcinoembryonic antigen (CEA), squamous cell carcinoma antigen (SCC), and p53, which are now clinically used as ESCC tumor markers, are inadequate for identifying subclinical patients with early tumors and predicting disease recurrence [6].

Noncoding RNAs (ncRNAs), such as microRNAs, small interfering RNAs, and long noncoding RNAs (lncRNAs), play important regulatory roles in the development of many diseases especially in cancers [7, 8]. The lncRNAs, defined as RNA molecules >200 nucleotides in length, have been shown to be involved in the regulation of gene expression in both tumor-suppressive and oncogenic pathways at epigenetic level, transcriptional level, and posttranscriptional level [9, 10]. Mounting evidence supported the potential markers of lnvRNAs in tumor diagnosis and prognosis. For instance, increased expression of BC200RNA has recently been proposed as a potential novel molecular marker for breast cancer [11, 12], while MALAT-1 gene overexpression suggests poor prognosis in lung cancer patients [13]. However, lncRNA expressions in blood have not been investigated as potential novel biomarkers for ESCC diagnosis or prognosis.

HOX antisense intergenic RNA (HOTAIR), a lncRNA localized on chromosome within the homeobox C (HOXC) gene cluster [14] was initially discovered as a repressor of the HOXD genes [15]. Recently, researchers found that HOTAIR was significantly overexpressed in a variety of tumors and was able to induce the proliferation and metastasis of these tumors [16]. Our previous study [17] found that high expression levels of HOTAIR correlated clinically with ESCC progression. Moreover, HOTAIR contributes to the malignant phenotype of ESCC cells through its regulation of diverse cellular processes, including migration, invasion, and proliferation [17]. So, this study aimed to investigate the expression of serum HOTAIR in ESCC patients and to evaluate its diagnostic value in the early diagnosis of ESCC.

## Methods

### Patients and blood samples

A total of 50 consecutive patients with ESCC were recruited at the Sun Yat-Sen Memorial Hospital of Sun Yat-Sen University from June 2013 to January 2016. All of these included subjects were newly diagnosed and previously untreated (including surgery, chemotherapy, and radiotherapy). The data including age, gender, histologic grade, T stage, lymph node metastasis, and TNM stage were recorded. Tumors were staged according to the TNM staging system of the American Joint Committee on Cancer (AJCC) [18]. As a control, 20 individuals who sought a routine health check-up at the same period and did not have any esophageal diseases or other cancerous diseases were recruited. All blood samples were collected with informed consent and agreement, according to protocols approved by the Ethics Committee of the Sun Yat-Sen Memorial Hospital of Sun Yat-Sen University.

Of those 50 patients with ESCC, 42 underwent surgery, and 8 did not undergo surgery because of distant metastasis. Postoperative venous blood samples from 42 patients were collected 1 month after the surgery. Preoperative venous blood samples of 50 ESCC patients and 20 healthy controls were also collected. Blood samples were centrifuged at 2000 r/min for 10 mins at 4 °C, and the serum was collected and stored at -80 °C until further processing. Of the 42 patients who underwent surgery, 20 ESCC tissues were collected during the operation. Fresh tissue samples were frozen within 30 min after surgery and stored in liquid nitrogen. Tissue sections from each ESCC sample were reviewed and classified by a pathologist.

### RNA extraction, reverse transcription, and quantitative real-time PCR(qRT-PCR)

Total RNA was extracted from each serum and ESCC tissue sample using Trizol LS reagent (Invitrogen life technologies, USA) and then reverse-transcribed with a SuperScriptTM III Reverse Transcriptase (Invitrogen) according to the manufacturer’s instructions. After that, qRT-PCR was performed to quantify the expression levels of lncRNAs using Gene Amp PCR System 9700 (Applied Biosystems, CA, USA) and the 2X PCR Master mix (KANGCHENG, Shanghai, China). The HOTAIR qPCR primers used are forward,5’-GGAAAGATCCAAATGGGACCA-3’; reverse,5’-CTAGGAATCAGCACGAAGCAAA-3’. U6 was evaluated as a housekeeping gene for the qPCR reactions. The U6 qPCR primers used are 5’-GCTTCGGCAGCACATATACTAAAAT-3’; reverse,5’-CGCTTCACGAATTTGCGTGTCAT-3’. The expression level of HOTAIR in each sample was normalized to that of the internal control U6. HOTAIR absolute expression levels were calculated by the the2 − ΔΔCt method.

### Statistical analysis

SPSS Statistics software version 17.0 was used for the statistical analysis. All data was analyzed by normal distribution test. Mean ± standard deviation (SD) was used to represent measurement data with normal distribution. Comparison of measurement data of normal distribution was analyzed by student’s *t*-test. Pearson correlation analysis was used to assess the association between two continues variables. Receiver operating characteristics (ROC) curve was plotted to determine how well the expression level of serum HOTAIR discriminated between tumor samples and healthy control samples. An AUC-ROC value of >0.7 was taken to indicate reasonable biomarker performance. ROC curves optimal cut-off values were defined as the point that maximized the Youden index, defined as (sensitivity + specificity)-1. A *P*-value of <0.05 was considered statistically significant.

## Results

### Clinicopathological characteristics and serum HOTAIR expression levels of ESCC patients

As shown in Table 1, the expression level of serum HOTAIR was closely associated with TNM stage (*P* = 0.002). Furthermore, patients with distant metastasis showed significantly higher HOTAIR expression level than patients without distant metastasis (0.248 ± 0.028 versus 0.189 ± 0.010, *P* = 0.004). However, there was no correlation detected in the expression level of serum HOTAIR with age, gender, lymph node metastasis, depth of invasion and degree of tumor differentiation.

**Table 1:** Clinicopathological characteristics and serum HOTAIR expression levels of ESCC patients

Variables	No. of patients	Serum HOTAIR expression (mean ± SD)	*P* value
Age, year			0.739
≥ 60	23	0.192 ± 0.014	
< 60	19	0.185 ± 0.015	
Gender			0.820
Male	33	0.190 ± 0.013	
Female	9	0.184 ± 0.013	
TNM stage			0.002
I	6	0.134 ± 0.050	
II	10	0.181 ± 0.051	
III	26	0.204 ± 0.069	
IV	8	0.261 ± 0.022	
T stage			0.140
T1-T2	11	0.144 ± 0.075	
T3	31	0.174 ± 0.083	
N stage			0.053
N0	15	0.160 ± 0.055	
N1	20	0.181 ± 0.061	
N2-N3	7	0.224 ± 0.037	
M stage			0.004
M0	42	0.189 ± 0.010	
M1	8	0.248 ± 0.028	
Differentiation			0.871
Well	12	0.197 ± 0.070	
Moderate	19	0.185 ± 0.061	
Poor	11	0.186 ± 0.061	

### The serum HOTAIR expression level of patients was positively correlated with the expression of HOTAIR in ESCC tissue

**Fig. 1:**
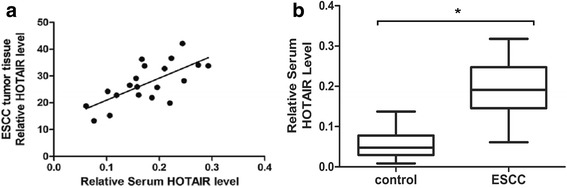
HOTAIR expression levels from different sources and patients. **a** *Pearson* correlation analysis of HOTAIR expression levels in ESCC tissue and serum; **b** Comparison of serum HOTAIR expression level between ESCC patients and healthy controls

HOTAIR expression levels in both ESCC tissues and serum of ESCC patients were analyzed to detect their relationship. As shown in Fig. 1a, we found that ESCC tissue had a significantly increased HOTAIR expression compared with the level in serum (*P* < 0.01). Also, serum HOTAIR expression level was positively associated with the expression of HOTAIR in ESCC tissue (*r* = 0.6904, *P* < 0.01). It suggested that serum HOTAIR might serve as a promising biomarker.

### Upregulation of serum HOTAIR level in ESCC patients

The expression level of serum HOTAIR in 50 ESCC patients and 20 healthy controls were detected to determine whether serum HOTAIR expression levels are higher in patients with ESCC. As shown in Fig. 1b, the serum of ESCC had a significantly increased HOTAIR expression compared with the level of healthy controls (0.189 ± 0.010 versus 0.055 ± 0.008, *P* < 0.01).

### ROC curve of serum HOTAIR level in the diagnosis of ESCC

**Fig. 2:**
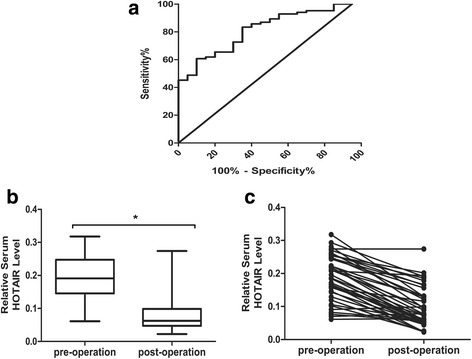
Serum HOTAIR levels as a diagnostic biomarker. **a** ROC curve analysis for serum HOTAIR in the diagnosis of ESCC; (**b**, **c**) Comparison of serum HOTAIR expression levels between preoperative and postoperative samples

We further analyzed ROC curve of serum HOTAIR level to assess its diagnostic value (Fig. 2a). We found that serum HOTAIR level could differentiate ESCC patients from healthy controls, with an AUC of 0.793 (95% CI: 0.692 to 0.895, *P* < 0.01). And the optimal cut-off values were 0.094 (sensitivity 56.0%, specificity 90.0%)

### The correlation of serum HOTAIR expression level between preoperative and postoperative patients in ESCC

The postoperative venous blood samples from 20 patients were collected 1 month after surgery. The expression level of serum HOTAIR in postoperative samples decreased significantly compared with the level of preoperative specimens (0.083 ± 0.008 versus 0.189 ± 0.010, *P* < 0.01) (Fig. 2b and c).

## Discussion

ESCC is an aggressive tumor with rapid growth rate and high opportunity of regional and distant metastasis, which contributes to the poor prognosis. Therefore, it is an urgent need to find an effective biomarker for ESCC so that the dismal tumor can be treated at the early stage. Circulating lncRNAs has been confirmed that they can be remarkably stable in serum and be detected in human peripheral blood in spite of the high amounts of RNase in the blood of cancer patients [19]. Clinically, overexpression of HOTAIR is a powerful predictor of overall survival and progression for several cancers including gastrointestinal stromal tumors [20], breast cancer [21], squamous cell carcinoma [22], nasopharyngeal carcinoma laryngeal [23], colon cancer [24], hepatocellular carcinoma [25, 26] and pancreatic cancer [27]. In our previous study, HOTAIR, a lncRNA has been considered as a prognostic marker for ESCC [17]. Here we found that the serum HOTAIR also showed great promise as a novel diagnostic biomarker of ESCC.

We measured the expression of HOTAIR in ESCC tissue and serum of 50 ESCC patients. The results revealed that ESCC tissue had a significantly increased HOTAIR expression compared with that of serum. Also, serum HOTAIR expression level was positively associated with the expression of HOTAIR in ESCC tissue. Combined with our previous study, it suggested that serum HOTAIR could serve as a promising biomarker. The qRT-PCR method was performed to examine the expression level of serum HOTAIR in 50 ESCC patients and 20 healthy controls to determine whether serum HOTAIR expression levels are higher in patients with ESCC, The results showed that serum of ESCC had a significantly increased HOTAIR expression compared with that of healthy controls. Moreover, we found that serum HOTAIR was significantly correlated with M stage and TNM stage. All these evidence further confirmed an important role of HOTAIR on the ESCC progression. ROC curve analysis showed serum HOTAIR could differentiate ESCC patients from healthy controls, with an AUC of 0.793. It revealed that serum HOTAIR could be used as a promising diagnostic indicator for patients with ESCC.

The release of nucleic acids into the blood is thought to be related to necrosis tumor cells from the tumor microenvironments [28]. Our results showed that the expression level of serum HOTAIR in postoperative samples decreased significantly compared with that of preoperative specimens. It revealed that the origin of HOTAIR derived from tumor cells and serum HOTAIR was down-regulated after the tumor resection. Therefore, serum HOTAIR expression level can serve as the indicator for cancer recurrence of ESCC patients who undergo tumor resection.

However, certain limitations in our study should be addressed as follows. The population of enrolled patients and controls was relatively small, which might result in bias in the final results. Besides, it is a pity that we could not gain enough data to perform survival analysis. Therefore, a longer follow-up study is needed to assess the prognostic significance of serum HOTAIR in ESCC.

## Conclusion

For the first time, the results of our study indicated that the expression of HOTAIR in serum could be used as a novel diagnostic biomarker for ESCC.
